# Multistage heat treatment and the development of a protective oxide-scale layer on the surface of FeCrAl sintered-metal-fibers

**DOI:** 10.1038/s41598-020-80888-9

**Published:** 2021-01-12

**Authors:** Osama M. Ibrahim, Abdullah A. Alazemi, Loai Ben Naji

**Affiliations:** 1 Mechanical Engineering Department, Faculty of Engineering and Petroleum, Kuwait University, Kuwait City, Kuwait; 2 Public Authority for Applied Education and Training, Kuwait City, Kuwait

**Keywords:** Materials science, Materials for energy and catalysis, Corrosion, Porous materials

## Abstract

This study investigates the effects of Multistage Heat Treatment (MSHT) on the development of an oxide-scale layer on the surface of FeCrAl sintered-metal-fibers. The oxide-scale layer was developed using an MSHT cycle at 930 °C for 1 h, followed by 960 °C for 1 h, and finally at 990 °C for 2 h. In this study, three samples were considered: Sample 1 was kept without thermal oxidation, while Samples 2 and 3 were exposed to one and eighteen MSHT cycles. Thermo-gravimetric analyses show that the weight gain of the heat-treated sample slows with time, confirming the growth of the protective oxide-scale layer. Scanning electron microscope images, after one MSHT cycle, reveal nonuniform oxide-scale growth with platelet-like on the surface. After eighteen MSHT cycles, however, clumped particles formed on the surface of the fibers. Atomic force microscopy was utilized to study the surface topography of the fibers. The results show that MSHT increases the surface roughness, where the surface roughness of one and eighteen MSHT cycles are the same. The x-ray diffraction analyses of the baseline sample and the sample with one MSHT cycle show pattern peaks of crystalline Fe_2_CrAl. In contrast, the results of eighteen MSHT cycles displayed diffraction pattern peaks of crystalline Cr and stable α-Al_2_O_3_. In summary, the results of this study reveal the changing nature of the oxide-scale layer. The findings of this study form the foundation for new techniques to protect and prepare the FeCrAl fibers as a support for catalysts.

## Introduction

The potential of high-temperature oxidation for protecting FeCrAl alloy base-metal from corrosion has received considerable attention. The high-temperature oxidation resistance of FeCrAl alloy is based on the formation of a dense and continuous protective aluminum oxide layer on the surface of the alloy when exposed to high temperatures. The oxidation layer on the FeCrAl surface is generally formed by single or multistage heat treatment (MSHT). FeCrAl alloy primarily consists of iron, chromium, and aluminum. A slight concentration of rare-earth elements such as yttrium, zirconium, and cerium is used to enhance the adhesion alumina on the surface of FeCrAl alloy base-metal ^1–6^. The study cited that the rare earth elements prevent oxygen grain boundary diffusion, and the Al_2_O_3_ scale growth rate can be decreased with increasing rare earth elements content. Naumenko et al. ^5^ studied the effect of combined yttrium and zirconium additives on the formation of the oxide scale on the surface of FeCrAl alloy. The thermal oxidation was carried out at temperatures between 1200 and 1300 °C in an argon-oxygen environment. Their results showed the effect of the zirconium on the texture of the Al_2_O_3_ scale on FeCrAl and the improvement of the oxide-scale adherence resulting in extending the lifetime of the base-metal alloy. Whittle and Stringer ^6^ studied the effect of reactive elements on the formation and adhesion of the oxide-scale layer at high-temperatures. They proposed several theoretical models to explain the improvement in oxidation resistance and the adhesion between the oxide-scale layer and the substrate alloy. Stott and Hiramatsu ^7^ studied thermal oxidation of thin foil made of FeCrAl alloy containing small concentrations of lanthanum or lanthanum plus molybdenum at 1150 °C. They concluded that thin foils of FeCrAl are inclined to breakaway oxidation due to the depletion of aluminum in the base metal alloy. A similar conclusion was reached by Naumenko et al. ^8^, where the high initial growth rate of the metastable oxide phases could lead to a critical depletion of the aluminum reservoir in thin-walled foil, resulting in breakaway failure.

FeCrAl alloy's resistance to high-temperature oxidation is based on the properties of the oxide-scale layer formed on the alloy surface. Thermal oxidation of FeCrAl alloy can also lead to interesting Al_2_O_3_ morphology formations with a high surface area such as platelets or whiskers ^7–23^. Pint et al. ^12^ study cited that the metastable chemical conversion, which contains most of θ-Al_2_O_3_, into stable α-Al_2_O_3_ leads to volume change. This volume change could come out with microcracks and peeling of the alumina scale from the FeCrAl alloy's surface during high-temperature oxidation cycles. The protective properties and durability of the desirable α-Al_2_O_3_ scale depend on the slow-growing rate, the concentration of the crystals phase in the alumina scale layer, and acceptable chemical stability in high-temperature corrosion environments. A novel accelerated MSHT process was patented by Vaneman and Sigler ^13^ for aluminum-containing stainless-steel alloys. They claimed that the novel process accelerates the growth rate and controls the shape of the growing oxide whiskers. Andrieu et al. ^14^ studied high-temperature oxidation between 850 °C and 1100 °C of thin FeCrAl strips. They presented a correlation between oxide growth kinetics, oxide morphology, and crystallographic structure. Badini and Laurella ^15^ performed a long-term thermal oxidation study (up to 30 days) on specimens of FeCrAl alloy at 900 °C and 1200 °C. Their results show that the oxide-scale layer was primarily made of α-Al_2_O_3_ with some chromium and iron. After the long-term testing, their results show that the aluminum content in the bulk alloy substrate was about 3%. They concluded that the treatment temperature controlled the growth rate of the oxide-scale layer, and the oxygen concentration in the gaseous atmosphere had a minor effect on the oxidation kinetics. Josefsson et al. ^16^ investigated the oxidation of FeCrAl alloys at 500 °C to 900 °C in dry oxygen. Their results show that the rate of oxidation increases with temperature. In a similar study, Liu et al. ^17^ investigated the formation of the alumina scales on FeCrAl alloy at 900 °C in dry oxygen or oxygen and water vapor atmosphere. Chevalier et al. ^18^ investigated the development of the alumina scales to identify the transient phases on the surface of FeCrAl alloy at 850 °C and 1100 °C for 100 h. Rallan and Garforth ^19^ studied the formation of metastable alumina phases on the surface of commercial FeCrAl alloy rods after thermal oxidation at 1000 °C for 8 h and 950 °C for 10 h. They used the oxide scale layer to anchor additional coatings of alumina as support for catalysts. Potter et al. ^20^ investigated the oxide-scale layer on the surface of thin foils of FeCrAl alloy. They observed the formation of voids under the oxide scale layer at times varying between 10 h at 900 °C to 10 min at 1200 °C. El-Kadiri et al. ^21^ studied single-stage thermal oxidation at 900 °C for 5 h on metal foils made of FeCrAl, which resulted in the formation of oxide nodules and crater on the foil surface. At the oxide crater nodule boundary, there was a radial arrangement of the platelets or whiskers. They reported that a change in the oxide volume causes tensile stresses, which led to the formation of cracks and affected the adhesion of the oxide-scale to the base metal alloy surface. The influence of aluminum concentration on the oxide-scale layer was investigated by Engkvist et al. ^22^. They considered six different FeCrAl alloys with aluminum concentration from 1.2% to 5.0%. The specimens were exposed to thermal oxidation at 900 °C for 72 h. Their results show that a minimum of 3.2% aluminum concentration is necessary to form a continuous protective oxide-scale layer. When the aluminum concentration is between 2 to 3%, they observed the formation of three separate oxide layers: an aluminum oxide-based inner layer, a chromium oxide-based intermediate layer, and an iron oxide-based outer layer.

The literature survey reveals many research papers that studied the formation of an oxide-scale layer on the surface of FeCrAl foils, strips, rods, or cuts of standard specimens. Few studies, however, considered FeCrAl fibers. Li et al. ^23^ and Fornasiero et al. ^24^ investigated the FeCrAl fibers as substrate and support of catalysts. Fei et al. ^25^ investigated the growth of metastable alumina platelets and whiskers on the surface of FeCrAl fibers during thermal oxidation temperatures between 800 °C and 900 °C. They reported that the rapid growth rate of the Al_2_O_3_ could deplete the aluminum content from the base metal alloy. They also investigated Titania coating to inhibit the growth of metastable alumina formation. An investigation by Samad et al. ^26^ showed the study outcomes of one cycle of the MSHT on FeCrAl fibers. The target of their study was alpha- Al_2_O_3_ formation for palladium catalyst support. The heat treatment process is carried out by step-up the oxidation temperature as follows, at 930 °C for 1 h, at 960 °C for 1 h, and 990 °C for 2 h for a total of 4 h. Their results showed that alpha-alumina is the major phase for the oxide scale on the surface of the fiber that is achieved by one cycle of MSHT. Although the MSHT process decreases the period for oxide scale growing on the FeCrAl fibers surface, it also decreases the aluminum reservoir, limiting the lifetime of the base metal FeCrAl alloy ^13,25,26^.

This study aims to investigate the effect of one and multiple MSHT cycles on the formation of an oxide-scale layer on the surface of FeCrAl sintered-metal-fibers. The MSHT heat treatment cycle by Samad et al. ^26^ was selected to accelerate the growth of the oxide-scale layer and observe its development. The development of the oxide-scale layer on the fibers' surface was studied using experimental techniques including SEM, EDS, TGA, XRD, and AFM to investigate the mechanism and experimental evidence for the high‐temperature oxidation on the surface of the fibers. The goal is to perform in-depth analyses to answer fundamental questions about the oxide-scale layer's evolution with repeatable heat treatment cycles.

## Motivation

**Figure 1:**
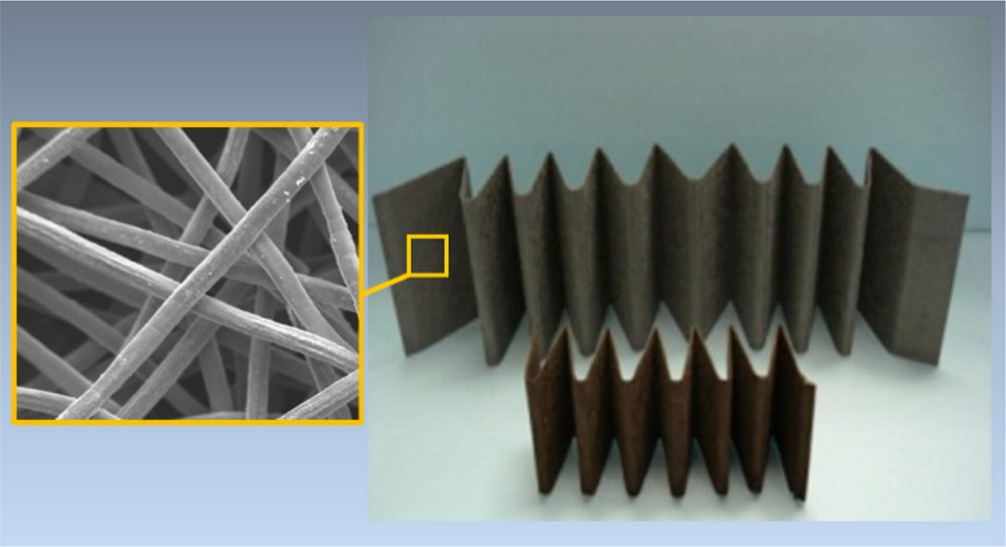
Pleated sintered-metal-fibers made of FeCrAl.

**Figure 2:**
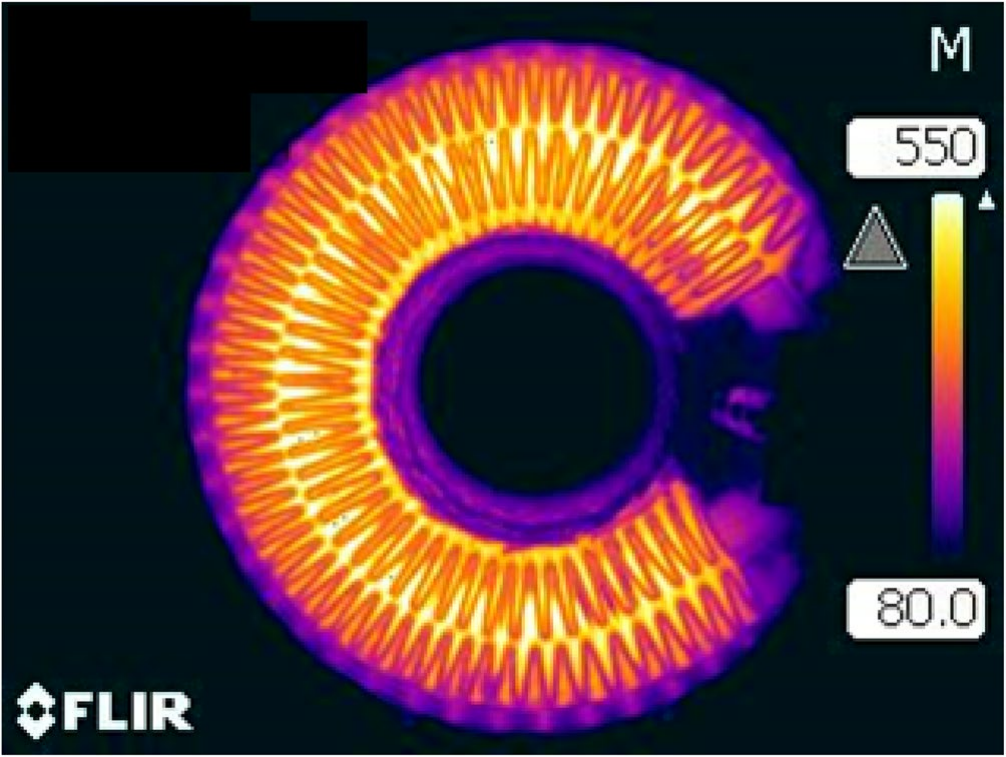
Thermal image of an electrically heated filter cartridge, high temperature of 550 °C.

This research focuses on the application of FeCrAl sintered-metal-fibers in high-temperature gas filtration using electrically heated filter cartridges that consist of strips of pleated sintered-metal-fibers, as shown in Fig. 1. The electrically conductive pleated strips are assembled into filter cartridges ^27,28^. Direct electrical heating is used to incinerate the trapped particulates in the filter medium. The electrical heating is cyclic and usually lasts for 2 to 5 min every hour. During a heating cycle, the filter medium can reach a maximum temperature between 500 °C to 850 °C, depending on the application and gas stream temperature. A thermal image of an electrically heated filter cartridge is shown in Fig. 2, where the maximum temperature of the filter medium approaches 550 °C. The hot gas and cyclic electrical heating expose the FeCrAl fibers to thermal oxidation. In this application, it is necessary to form a protective oxide-scale layer on the surface of the fibers that lasts throughout the life duration of the filter cartridge. FeCrAl is known to have excellent oxidation resistance and is usually used in the construction of components that operate at high temperatures. The oxidation resistance of FeCrAl alloy relies on the formation of a slowly growing Al_2_O_3_ layer that forms during high-temperature thermal oxidation.

### Sample preparation

Three samples of FeCrAl sintered-metal-fibers, 40 µm in diameter, were considered in this study. The samples were cleaned with alcohol in an ultrasonic bath for 30 min, then dried in an oven at 120 °C for 1 h. The first sample, Sample 1, was kept without heat treatment as a baseline, and the second sample, Sample 2, was heat treated using one MSHT cycle for 4 h. The third sample, Sample 3, was heat treated using eighteen MSHT repeated cycles of 4 h for a total of 72 h. The details of the MSHT cycle are summarized in Table 1, while the heat treatments of the three samples are summarized in Table 2. The chemical compositions of the tested FeCrAl fibers are shown in Table 3.

**Table 1:** Multi-Stage Thermal Oxidation (MSHT) cycle.

Heat Treatment Stage	Temperature (°C)	Time (hour)
1	930	1
2	960	1
3	990	2

**Table 2:** Heat treatment of the three sintered-metal-fibers samples.

	Heat treatment cycle/time	Number of cycles
Sample 1	No heat treatment	0
Sample 2	MSHT/4 h	1
Sample 3	MSHT/72 h	18

**Table 3:** The elemental chemical compositions of the FeCrAl sintered-metal-fibers.

Element	Composition (%)
Cr	20.580
Al	5.760
Mn	0.160
Cu	0.046
Ti	0.041
C	0.033
P	0.015
S	0.002
N	0.010
Si	0.240
Fe	Balance

## Results and discussion

### Thermo-gravimetric analysis

**Figure 3:**
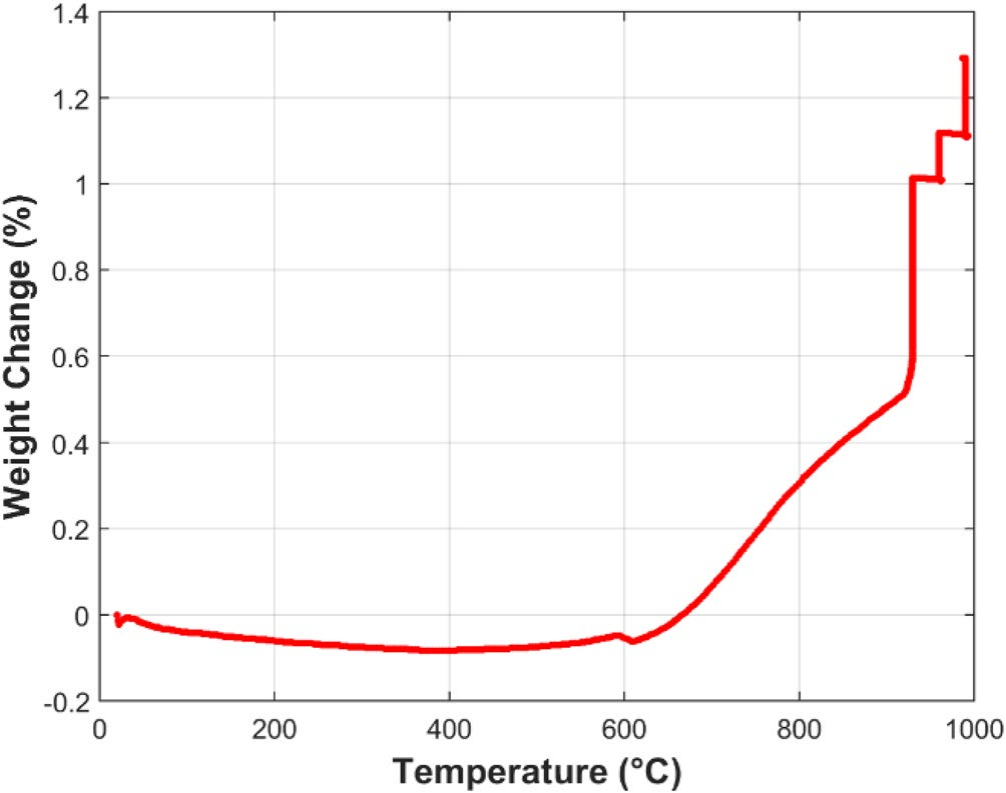
Percentage weight change of the FeCrAl fibers versus temperature during the first MSHT cycle at 50 °C/min heating rate between set temperatures.

**Figure 4:**
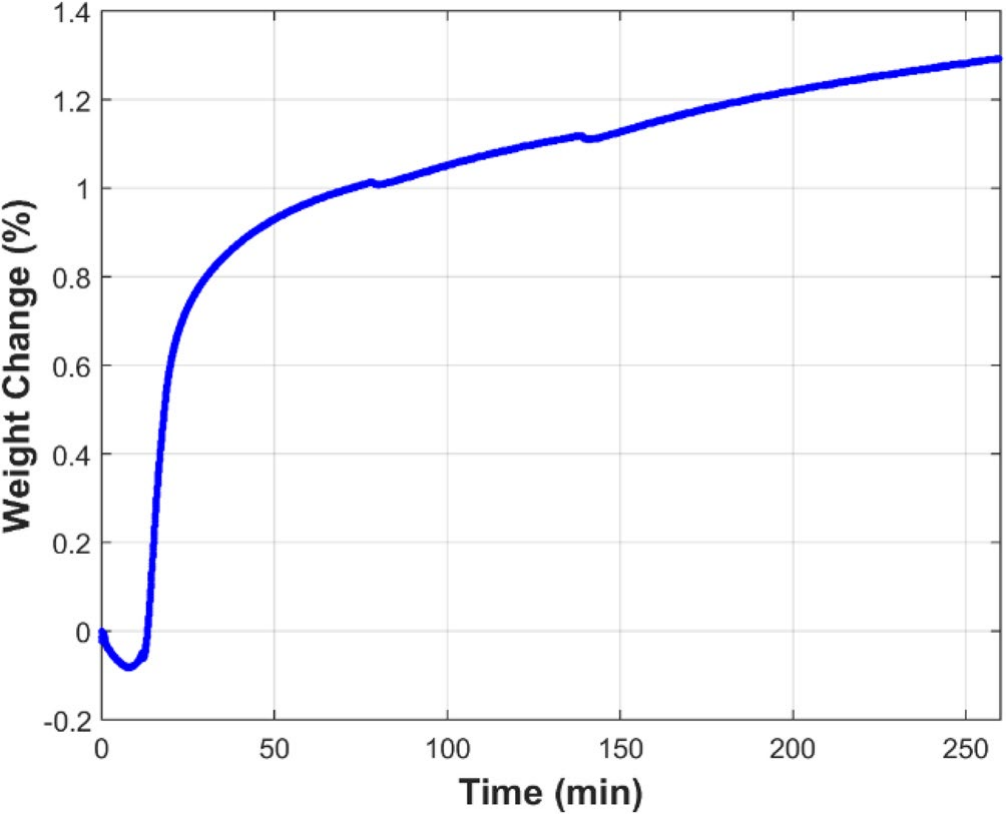
Percentage weight change of the FeCrAl fibers versus time during the first MSHT cycle.

**Figure 5:**
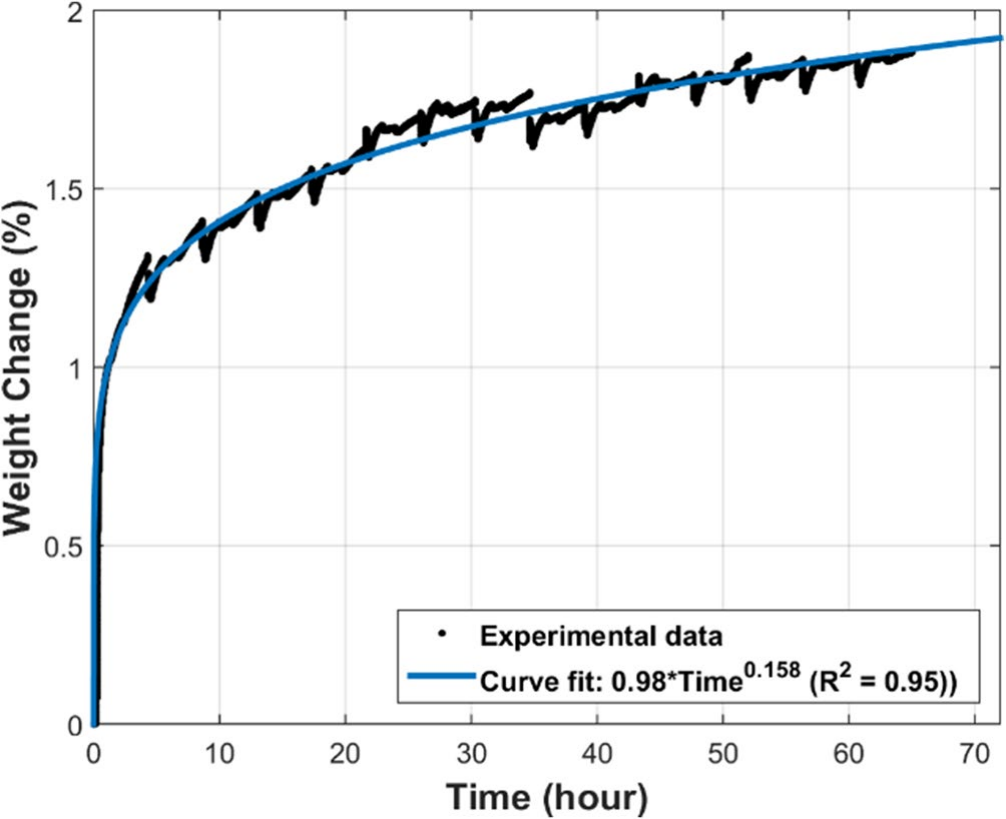
Percentage weight change of the FeCrAl fibers versus time during multiple MSHT cycles.

Thermo-Gravimetric Analysis (TGA), using TA Instruments TGA Q50 analyzer, was conducted to measure the mass variation of the FeCrAl fibers during the MSHT cycles. The TGA studies were performed to quantify the development of the oxide-scale layer on the surface of the fibers. Figure 3 shows the TGA curve of the FeCrAl sintered-metal-fibers during heat treatment at a 50 °C/min heating rate between the set temperatures. Initially, the temperature was ramped up from 25 to 930 °C, marking the start of one MSHT cycle, as summarized in Table 1. The weight loss at the beginning of the heating process, up to 390 °C, as shown in Fig. 3, is attributed to the evaporation of adsorbed gases and moisture in the sample. The TGA curve demonstrates that the FeCrAl fibers gained weight as the temperature increased from 600 °C to 990 °C. This increase in weight is due to the formation of the protective oxide-scale layer on the fibers by thermal oxidation. The weight gain from 1-h oxidation at 930 °C is significantly more than the weight gain from 1-h oxidation at 960 °C or 2-h oxidation at 990 °C. Figure 4 depicts the percentage weight change of the FeCrAl fibers versus time for one complete MSHT cycle. The weight increase in the first 20 min, and just after reaching the first setup point of 930 °C, was 0.64%; while a weight increase of 0.40% during the 1 h at 930 °C, 0.10% during the 1 h at 960 °C, and 0.19% during 2 h at 990 °C, were observed during the first MSHT cycle. Figure 5 illustrates the percentage weight change of the FeCrAl fibers versus time for multiple cycles of MSHT. The trend of percentage weight change versus time of all MSHT cycles was similar, but the amount of weight gain is different. The first MSHT cycle has the most significant increase in weight compared to the subsequent cycles. The relationship between the percentage weight gain and time is found to be a power curve, as shown in Fig. 5. The slowdown of the weight gain could be attributed to the formation of the protective oxide-scale layer on the surface of the fibers, which hinders oxygen from reaching the surface of the fibers and hence diminishes the oxidation process.

### Scanning electron microscope micromorphology and energy dispersive spectroscopy

The micromorphology of the FeCrAl fibers was examined using a field-emission Scanning Electron Microscope (SEM), model JEOL JSM-7001F, operating at an acceleration voltage of 25 kV to obtain high-resolution images of the surface. Chemical analyses of the surface of the fibers were performed using Energy Dispersive x-ray Spectroscopy (EDS). The three samples described in Table 2 above were investigated in this study.

#### Baseline—before heat treatment—as received

**Figure 6:**
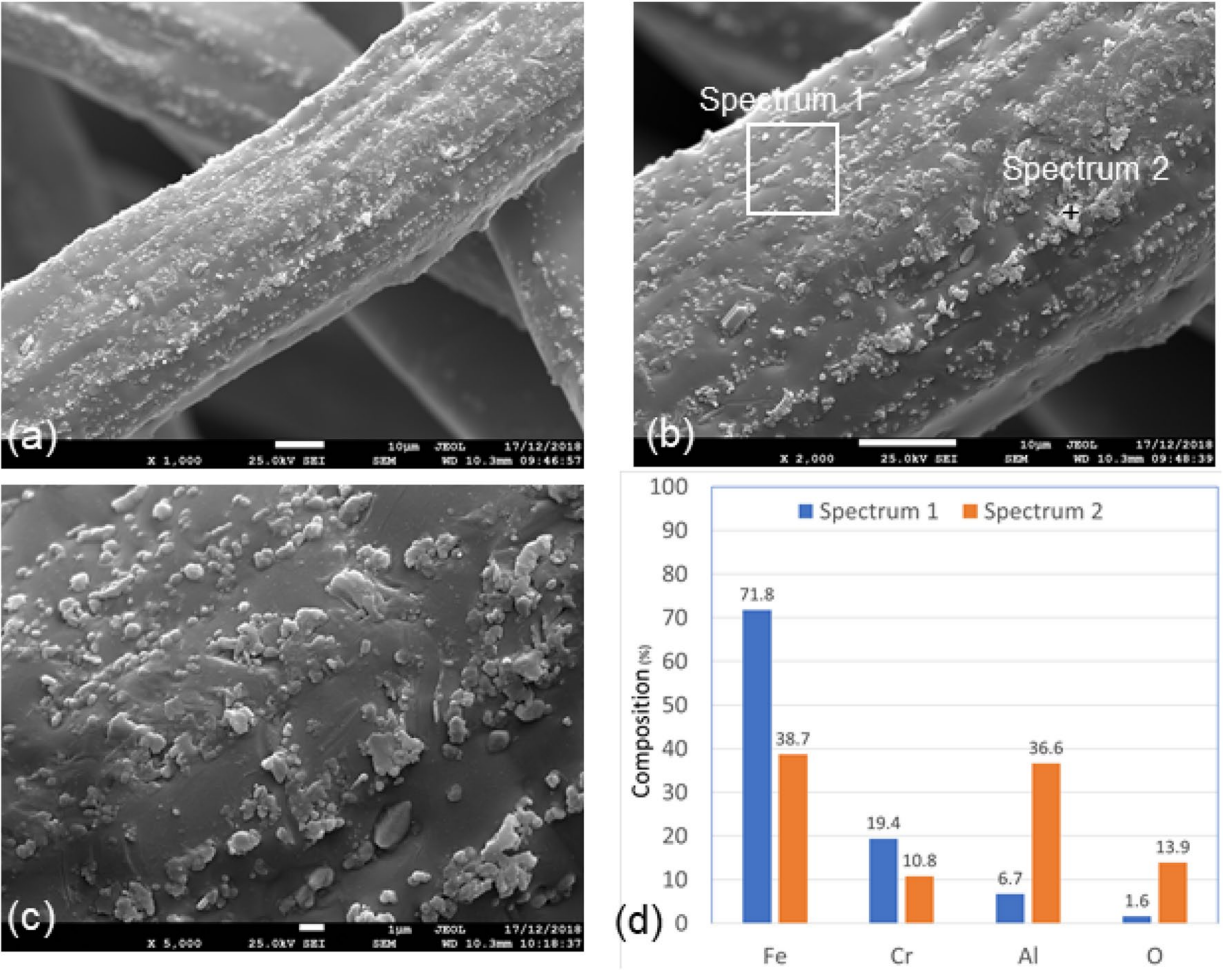
SEM micrograph images and EDS analysis of Sample 1, without heat treatment–as received, FeCrAl fibers at different magnifications, (**a**) × 1000; (**b**) × 2000; (**c**) × 5000; (**d**) EDS scans of Spectrum 1 and Spectrum 2.

Figures 6(a-c) present three SEM micrograph images of different magnifications of Sample 1. Sample 1 micrograph images show nonuniform distributions of nodules all over the fibers. The EDS analyses of Sample 1 shows that there is a variation in the elemental composition between Spectrum 1 and Spectrum 2. Spectrum 1 represents scan of a selected area, including some nodules, while Spectrum 2 represents a spot scan of a nodule on the surface of the metal fiber. The results of the EDS analyses, presented in Fig. 6d, show that Spectrum 1 has an elemental composition of 71.8% iron, 19.4% chromium, and 6.7% aluminum, which are approximately similar to the base metal alloy shown in Table 3. The results of Spectrum 2, however, show that the nodule has an aluminum content of 36.6% and oxygen contents of 13.9%, which are much higher than the average aluminum and oxygen contents on the surface of the fiber.

#### Sample 2: after one MSHT cycle

**Figure 7:**
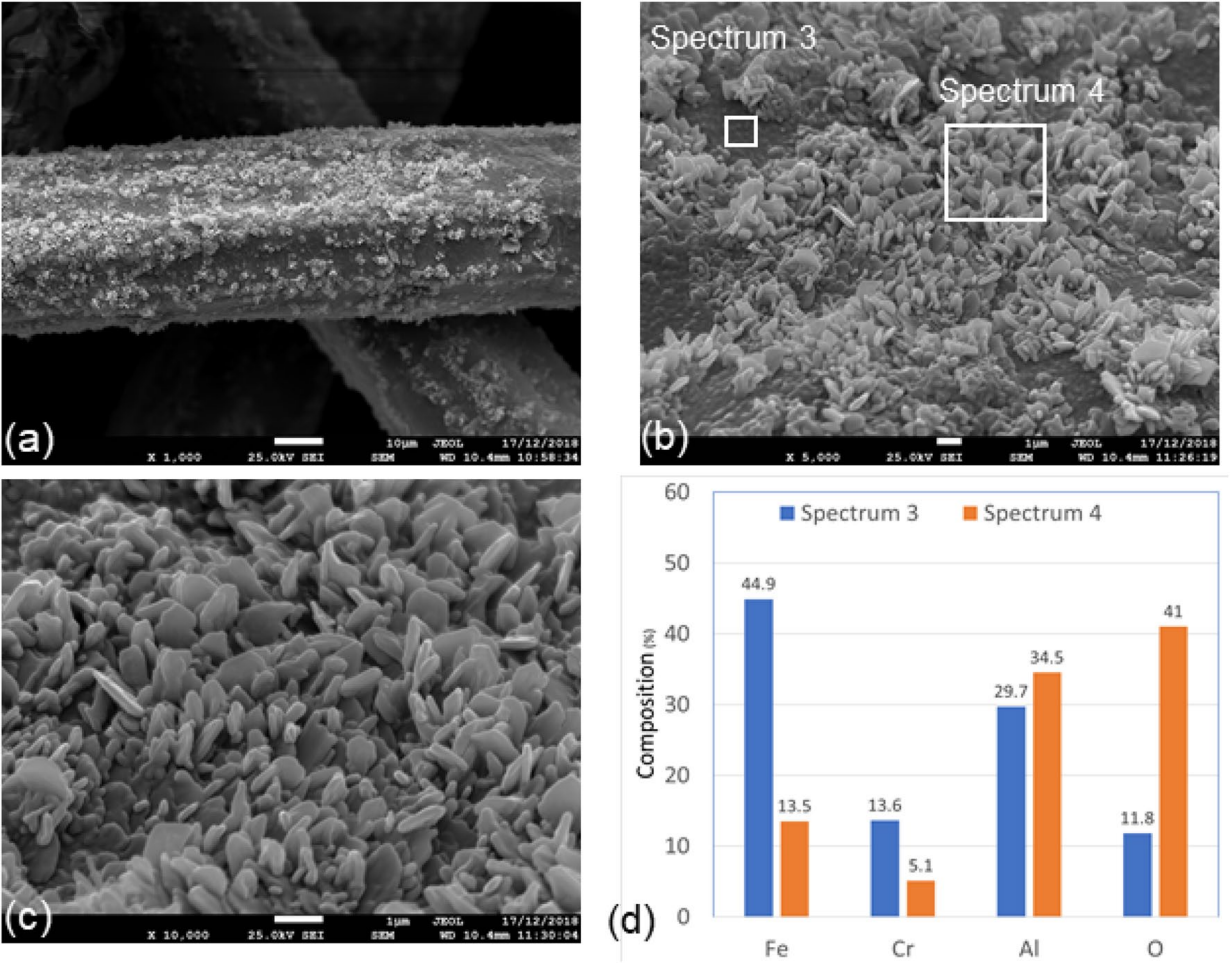
SEM micrograph images and EDS analysis of Sample 2, after one MSHT cycle, FeCrAl fibers at different magnifications, (**a**) × 1000; (**b**) × 5000; (**c**) × 10,000; (**d**) EDS scans of Spectrum 3 and Spectrum 4.

Figures 7(a-c) show three SEM micrograph images of different magnifications of Sample 2 with nonuniform morphology of the oxide-scale layer. The micrograph images show a forest with platelet-like morphology. They also show nonuniform surface morphology, with gabs displaying relatively a smooth oxide-scale layer with no distinct features on the surface. Elemental compositions of Spectrum 3, for an area with a smooth oxide-scale layer, and Spectrum 4, for a forest with platelet-like morphology are displayed in Fig. 7d. Compared to Spectrum 3, Spectrum 4 shows more aluminum and oxygen and less iron and chromium. The results indicate there is nonuniform aluminum diffusion on the surface of the fibers. Furthermore, the elemental compositions of Spectrums 3 and 4 show aluminum contents of 29.7% and 40.5% and oxygen contents of 11.8% and 28.2%, which are higher compared to the elemental chemical composition of the base metal shown in Table 3. The results confirm the formation of the Al_2_O_3_-base protective layer on the fibers by the multistage thermal oxidation.

**Figure 8:**
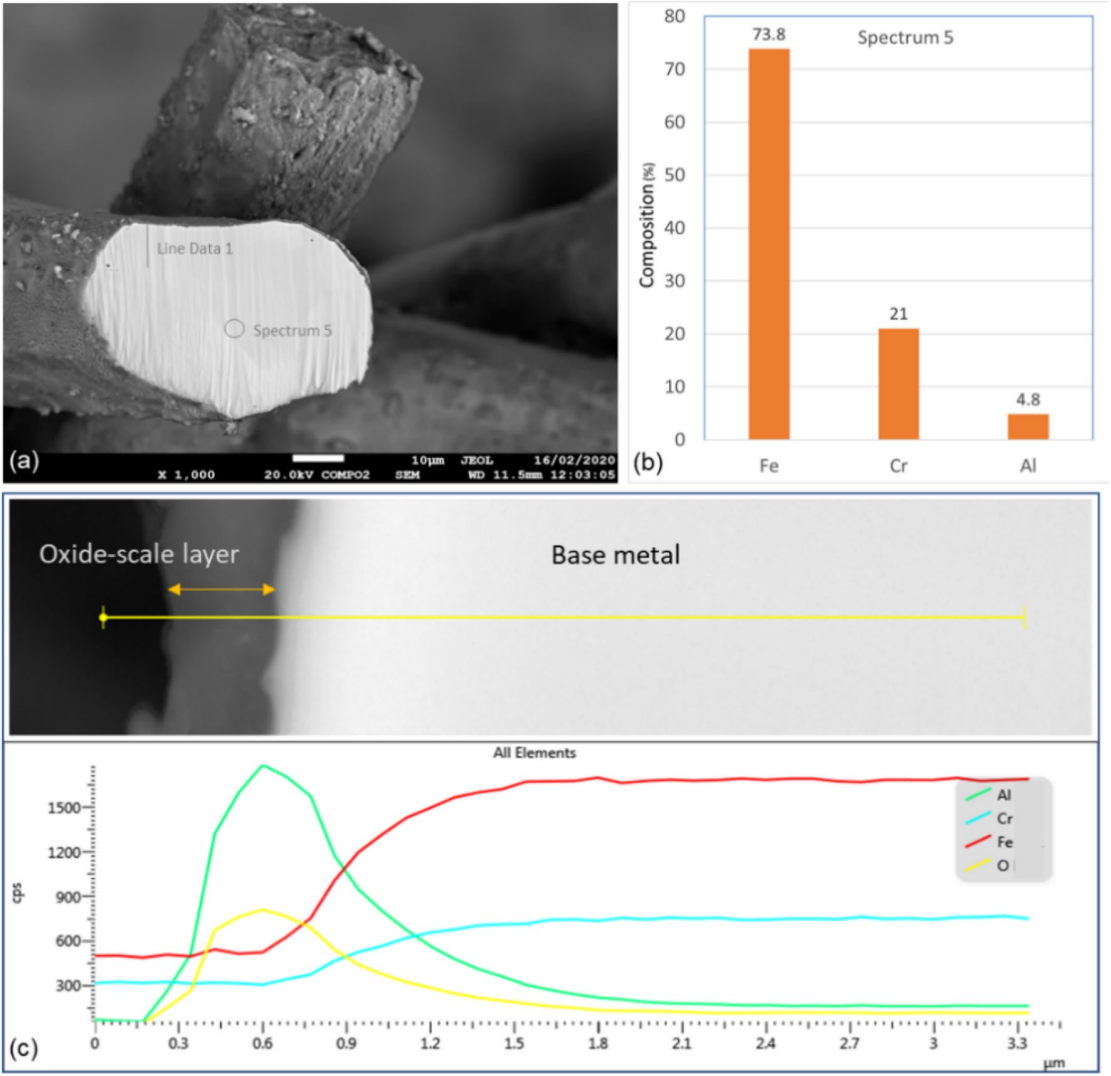
(**a**) Cross-section of Sample 2, 40 µm fiber; (**b**) EDS scans of Spectrum 5; (**c**) The characteristic x-ray counts along the specified line scan (Line Data 1) marked in Fig. 8a.

A cross-section polisher (JEOL-SM-09010) was used to prepare cross-sections of Sample 2 fibers for SEM and EDS analyses. The sample was placed in the holder, and an optical microscope was used to select the spot to be irradiated by the argon-ion beam. A high-quality cross-section of the 40 μm fiber, shown in Fig. 8a, was then chosen for further analysis. EDS scans were performed on a circular area approximately on the center of the fiber and along a line starting from the outer edge of the oxide-scale layer into few microns of the base-metal. The results of the EDS scan of the circular area, Spectrum 5, are shown in Fig. 8b. After one MSHT cycle, the aluminum composition in the center of the fiber is slightly less than that of the FeCrAl elemental composition shown in Table 3, indicating limited diffusion of aluminum from the base metal to the surface of the fiber.

**Figure 9:**
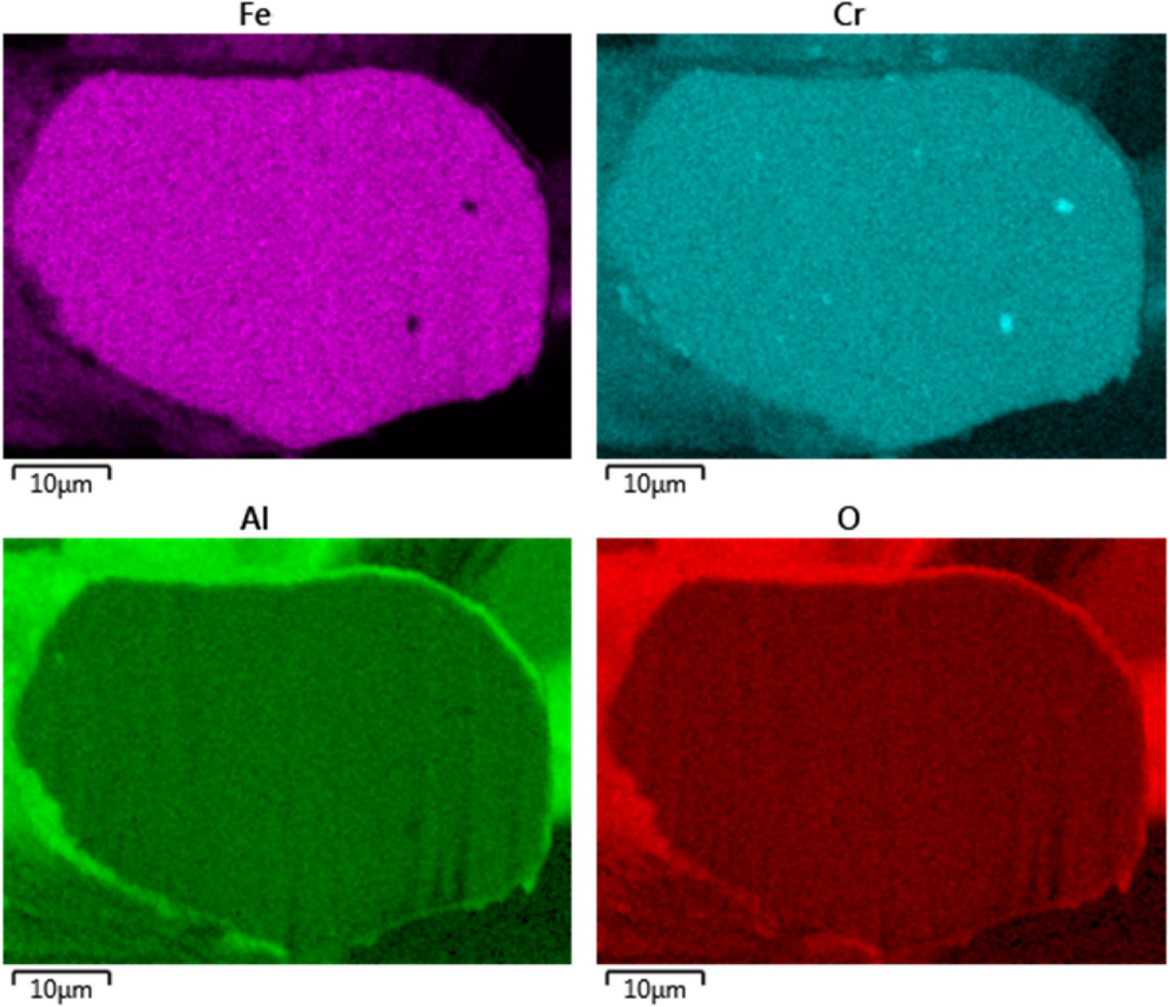
EDS mapping of iron, chromium, aluminum, and oxygen of Sample 2.

The x-ray EDS line scan count data in Fig. 8c show high concentrations of aluminum and oxygen and low levels of iron and chromium on the oxide-scale layer compare to the base-metal. After one MSHT cycle, the thickness of the oxide-scale layer varies from 0.4 μm to 0.6 μm. Figure 9 shows x-ray maps for iron, chromium, aluminum, and oxygen. The results of the aluminum and oxygen x-ray maps confirm the formation of a thin layer of Al_2_O_3_ on the surface of the fiber. The x-ray maps of iron, chromium, and aluminum show a homogeneous FeCrAl alloy, except for few spots with high chromium concentration, protected by a thin oxide-scale layer dominated by Al_2_O_3_.

#### Sample 3: after eighteen MSHT cycles

**Figure 10:**
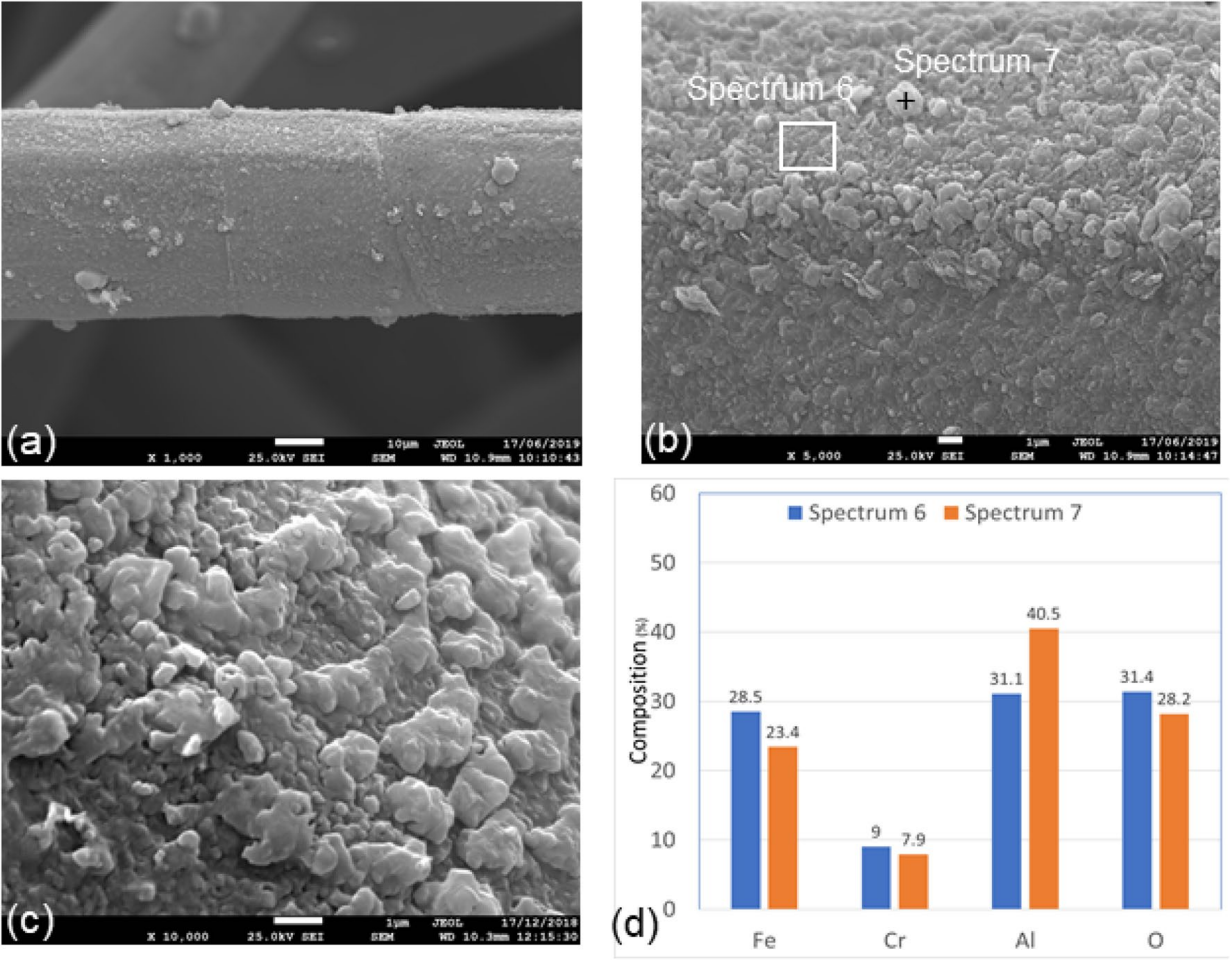
SEM micrograph images and EDS analysis of Sample 3, after eighteen MSHT cycles, FeCrAl fibers at different magnifications, (**a**) × 1000; (**b**) × 5000; (**c**) × 10,000; (**d**) EDS scans of Spectrum 6 and Spectrum 7.

Figures 10(a-c) show three SEM micrograph images of different magnifications of Sample 3 with a relatively uniform oxide-scale layer with clumped oxide particles on the surface of the fiber. Elemental compositions of Spectrum 6 and Spectrum 7 are displayed in Fig. 10d. Spectrum 6 represents scan of a selected area on the surface of the fiber, including some clumped particles, while Spectrum 7 represents a spot scan of a clumped particle. The aluminum content of Spectrum 7 is higher compared to Spectrum 6, 40.5% compared to 31.1%. The iron, chromium, and oxygen contents, on the other hand, show approximately the same compositions. The results indicate that after eighteen MSHT cycles, there is continuous aluminum diffusion on the surface of the fibers, which feeds the formation of the protective layer on the fiber surface and makes it more uniform. When we compare Fig. 10c with Fig. 7c, the oxide-scale layer was evolving and changing with the MSHT cycles from platelet-like and whiskers morphology to larger clumped particles.

**Figure 11:**
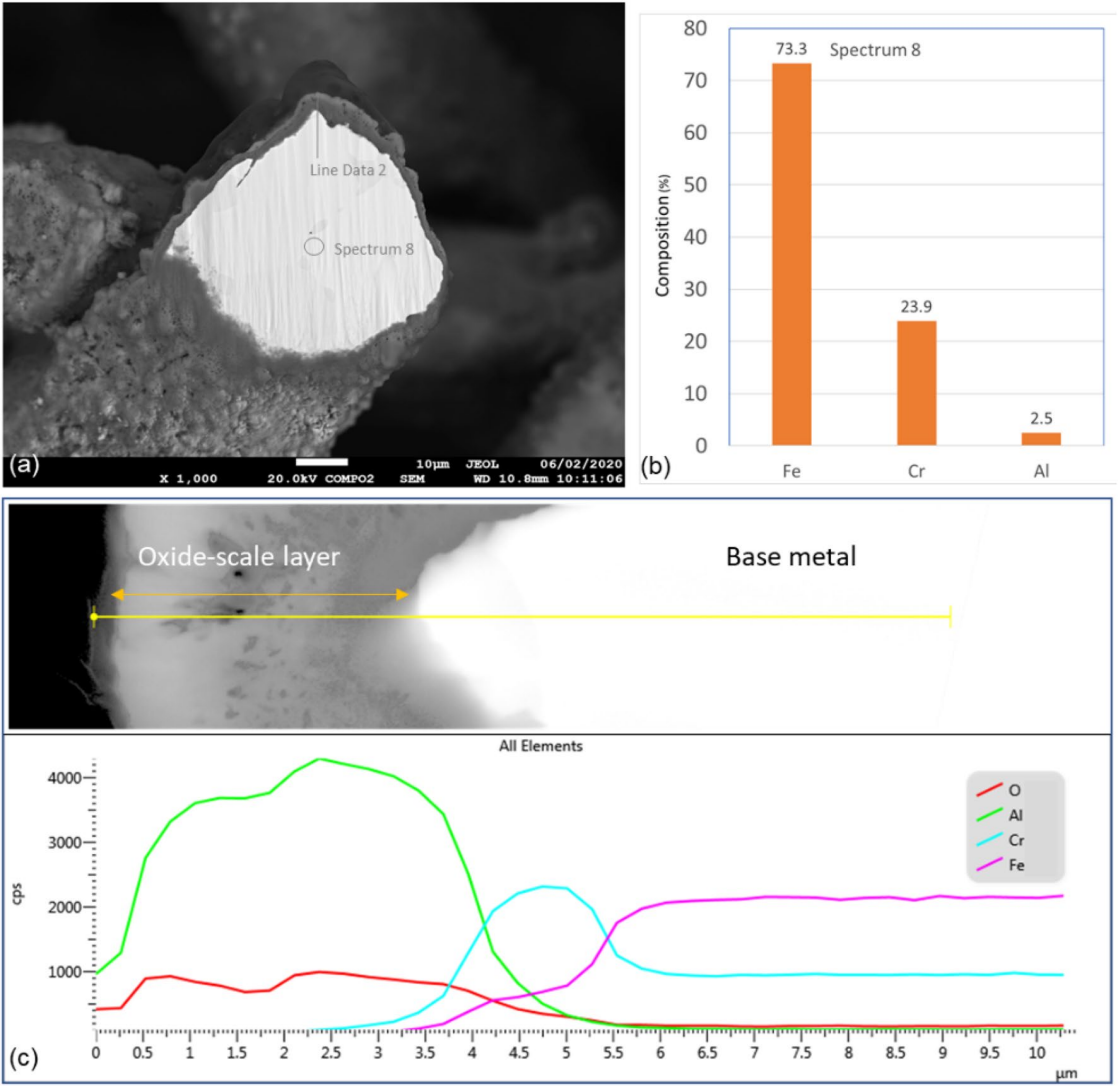
(**a**) Cross-section of Sample 3, 40 µm fiber; (**b**) EDS Scan of Spectrum 8; (**b**) The characteristic x-ray counts along the specified line scan (Line Data 2) marked in Fig. 11a.

Like Sample 2, a high-quality cross-section of Sample 3 fiber, shown in Fig. 11a, was prepared and selected for further SEM and EDS analysis. EDS scans were performed on a circular area approximately on the center of the fiber and along a line starting from the outer edge of the oxide-scale layer into few microns of the base-metal. The results of the EDS scan of the circular area, Spectrum 8, are shown in Fig. 11b. The aluminum composition in the center of the fiber is about 2.5%, which is much less than the FeCrAl elemental composition shown in Table 3, indicating relatively high diffusion of aluminum from the base metal to the surface of the fiber.

**Figure 12:**
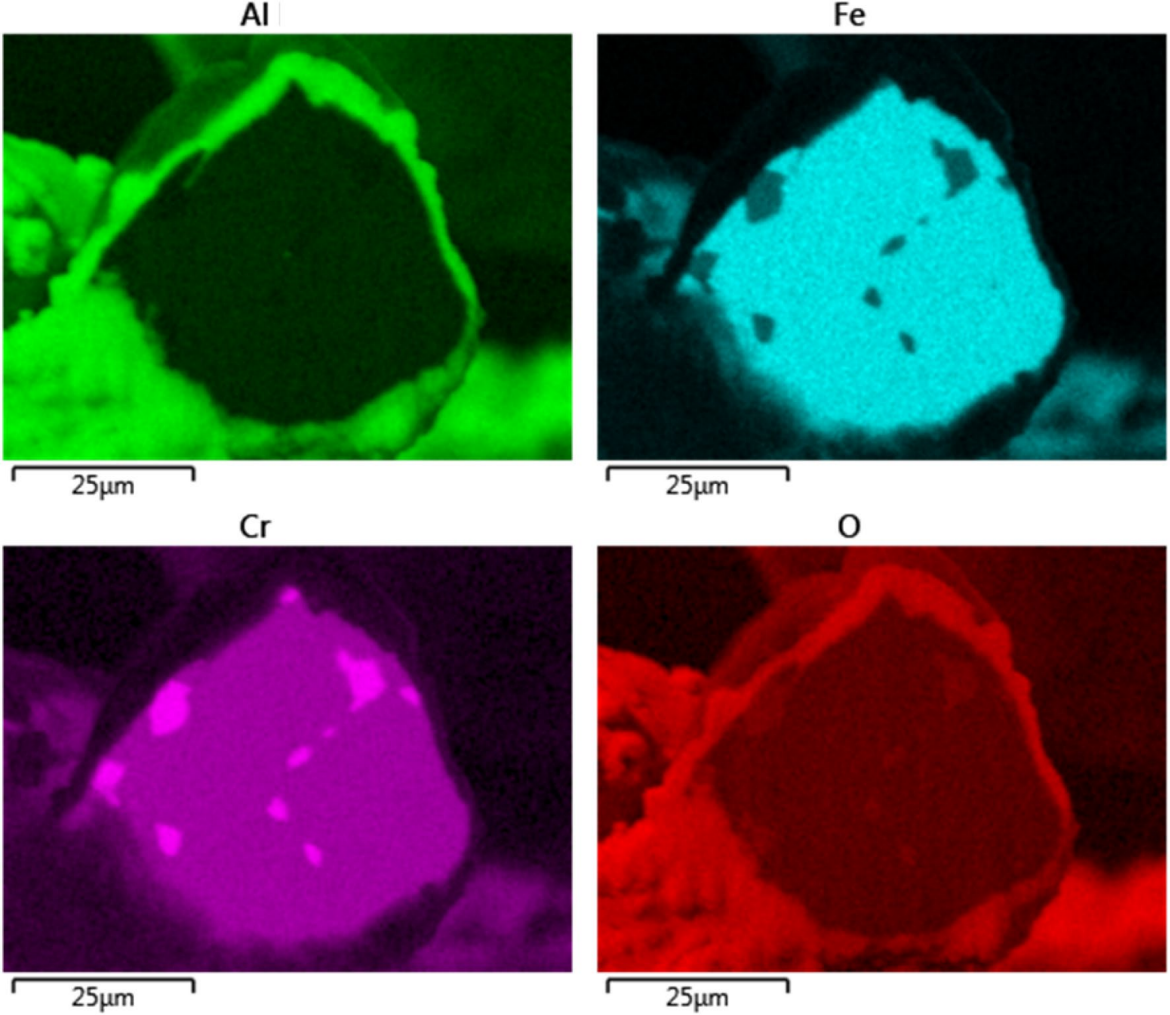
EDS mapping of iron, chromium, aluminum, and oxygen of Sample 3.

The x-ray EDS line scan count data in Fig. 11c show high concentrations of aluminum and oxygen and low levels of ion and chromium on the oxide-scale layer compare to the base-metal. After eighteen MSHT cycles, the thickness of the oxide-scale layer grew to 3.5—4.5 µm. Figure 12 shows x-ray maps for iron, chromium, aluminum, and oxygen. The results of the aluminum and oxygen x-ray maps confirm the formation of a thick layer of Al_2_O_3_ on the surface of the fiber. In contrast to Sample 2, the x-ray maps of iron, chromium, and aluminum of the base-metal show a non-homogeneous FeCrAl alloy with relatively large spots with high chromium concentration. This x-ray map results are also confirmed by the line scan count data in Fig. 11c, which also shows a high concentration peak of chromium coincides with low iron concentration.

### Atomic force microscopy

Agilent 5500 Atomic Force Microscopy (AFM) was utilized to study the effect of heat treatment on the surface topography of fibers. AFM is an instrument to produce a topographic image of solid samples with high resolution as well as to provide useful information on surface roughness parameters of the sample. In this study, AFM was used to obtain two-dimensional and three-dimensional topographical images of the fibers. Tapping-mode AFM scanning was conducted at room temperature using rectangular cantilever AFM probes (Nano-Sensors, tip radius less than 10 nm and a nominal length of 125 μm, mean width of 26 μm, and thickness of 4 μm). The three fiber samples were scanned.

**Figure 13:**
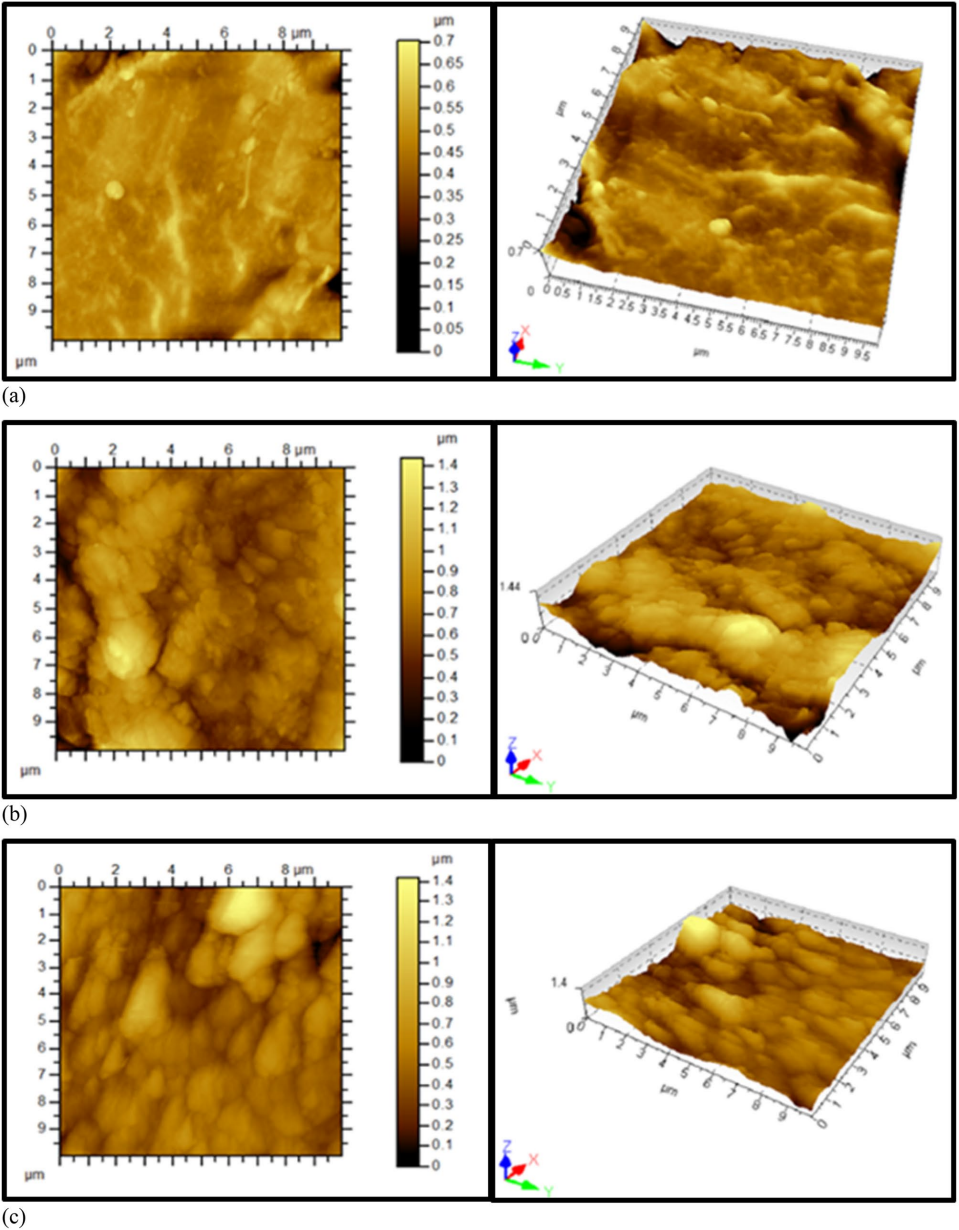
Two-dimensional (left) and three-dimensional (right) AFM images of FeCrAl fibers samples; (**a**) Sample 1 with no heat treatment; (**b**) Sample 2 after one MSHT cycle; (**c**) Sample 3 after eighteen MSHT cycles.

Figure 13 shows two-dimensional and three-dimensional AFM images of the three fibers samples at different heat treatment conditions. The thermal oxidation of the fibers increases the surface roughness where the variation in the height of Sample 1 (0.7 μm) is half of the Samples 2 and 3 (1.4 μm). Table 4 summarizes the surface roughness parameters obtained from AFM images for the three samples. The thermal treatment led to an increase in surface roughness, as measured by the root mean square (*R*_*q*_*)* and arithmetic mean *(R*_*a*_*)* of Samples 2 and 3 compared to Sample 1 by a factor of about 3. Interestingly, Sample 2 and Sample 3 have approximately the same surface roughness even though the duration of the heat treatment process was different.

**Table 4:** Surface roughness parameters obtained from AFM scanning the surface of fibers of the three samples.

	Sample 1	Sample 2	Sample 3
Root mean square, *R*_*q*_ (μm)	0.06	0.16	0.17
Arithmetic mean, *R*_*a*_ (μm)	0.04	0.12	0.12

### X-ray diffraction analyses

**Figure 14:**
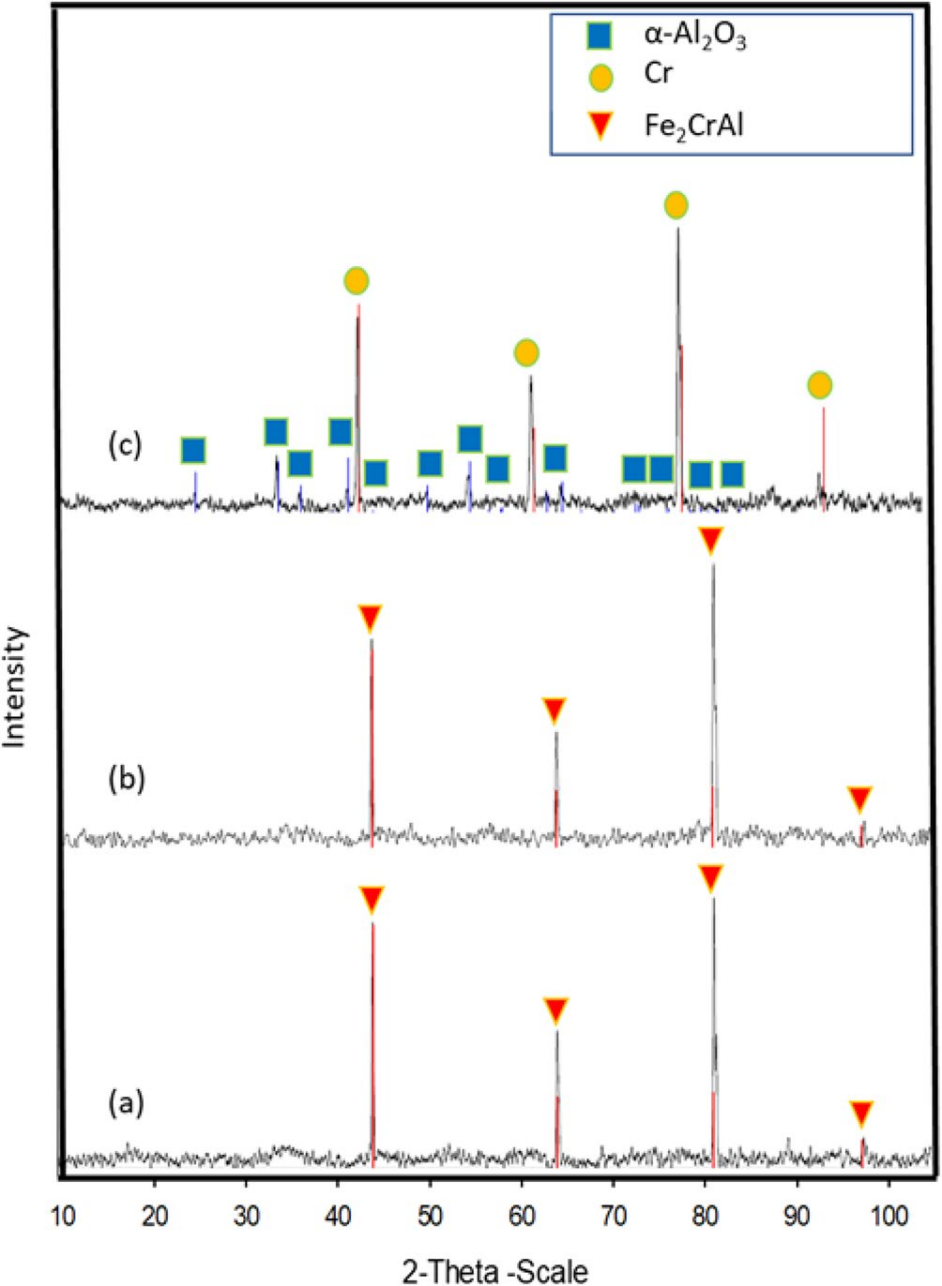
XRD test shows the crystalline pattern for (**a**) Sample 1, (**b**) Sample 2, and (**c**) Sample 3.

X-Ray Diffraction (XRD) analyses were performed to identify the crystalline formation on the surfaces of the FeCrAl fibers of the three samples. Figure 14 shows 2-dimensional diffraction patterns of the surface of the sample before oxidation, after one MSHT, and after 18 MSHT. The diffraction pattern peaks of Samples 1 and 2 clearly identify the crystalline Fe_2_CrAl phase formation. The stable α-Al_2_O_3_ diffraction pattern peaks were not detected; this indicates diffusion of the alumina layer presents as an amorphous formation on the fiber's surface. In contrast, Sample 3 results showed clearly diffraction pattern peaks of crystalline Cr and α-Al_2_O_3_, which means corundum nucleation occurs after multiple MSHT cycles. Compared to Sample 1 and Sample 2, Sample 3 results do not show the diffraction pattern peaks of crystalline Fe_2_CrAl. This result can be explained by the formation of the thick layer of the dense crystalline α-Al_2_O_3_ phase on the surface of the FeCrAl Fibers after eighteen MSHT cycles.

## Concluding remarks

In this study, we investigated the effect of MSHT on the formation and development of the protective oxide-scale layer on the surface of sintered-metal-fibers made of FeCrAl alloy. TGA was performed to quantify the weight change during the formation of the oxide-scale layer. The heat-treated sample gained weight, where the percentage weight increase diminishes with time and the rise in the number of MSHT cycles. The slowdown of the weight gain could be attributed to the formation of the protective oxide-scale layer on the fibers' surface, which hinders oxygen diffusion from reaching the surface of the fiber and hence reduces the thermal oxidation.

SEM micromorphology and EDS spectroscopy were used to study the growth of the oxide-scale protective layer. The thermal oxidation of FeCrAl alloy microfibers, after one MSHT cycle, results in the formation of predominantly Al_2_O_3_ platelet-like formation on the surface. The SEM images further reveal that the oxide-scale layer was not uniform on the surface of the fibers. However, after multiple MSHT cycles, a relatively uniform oxide-scale layer and clumped oxide particles were observed on the surface of the fibers. The results prove that the oxide-scale layer is evolving and changing with the multiple heat treatment cycles from platelet-like morphology to clumped particles. The AFM analyses of the fibers surface topography show that heat treatment increases the surface roughness where the surface roughness of one and eighteen MSHT cycles are approximately the same.

XRD analyses after one multistage thermal oxidation reveal the existence of amorphous alumina and diffraction pattern peaks of crystalline Fe_2_CrAl. In contrast, the results of eighteen multistage thermal oxidation cycles displayed diffraction pattern peaks of crystalline Cr and stable α-Al_2_O_3_.

The x-ray maps of the fiber cross-section after one MSHT show a relatively homogeneous base-metal alloy with few tiny spots of high chromium concentration. However, after eighteen MSHT cycles, the x-ray maps show a non-homogeneous base-metal alloy with relatively large spots with high chromium concentration, resulting from the multiple MSHT cycles and the high diffusion of the aluminum.

It is known that the multistage thermal oxidation accelerates the growth rate of the Al_2_O_3_ protective layer. The rapid growth rate and the phase transformation of the Al_2_O_3_ layer can cause internal stresses due to the variation in volume and thermal expansion, leading to cracks and spalling of the protective oxide-scale layer. EDS spectroscopy of the fibers' surface after heat treatment shows high aluminum and oxygen contents, confirming the formation of the oxide-scale layer. This study reveals and documents the changing nature of the oxide-scale layer with MSHT cycles, which should be considered when used as a carrier of additional wash-coats and catalysts.

## Data Availability

The datasets generated during and/or analyzed during the current study are available from the corresponding author on reasonable request.
